# Study on the Effect of Hydrothermal Carbonization Parameters on Fuel Properties of Chicken Manure Hydrochar

**DOI:** 10.3390/ma15165564

**Published:** 2022-08-13

**Authors:** Małgorzata Hejna, Kacper Świechowski, Waheed A. Rasaq, Andrzej Białowiec

**Affiliations:** Department of Applied Bioeconomy, Wrocław University of Environmental and Life Sciences, 37a Chełmońskiego Str., 51-630 Wrocław, Poland

**Keywords:** organic waste, waste to energy, waste to carbon, solid fuel, hydrochar, temperature, hydrothermal treatment

## Abstract

Economic development and population growth lead to the increased production of chicken manure, which is a problematic organic waste in terms of its amount, environmental threats, and moisture content. In this study, hydrothermal carbonization, an emerging way of waste disposal, was performed on chicken manure to produce an energy-rich material called hydrochar. The effects of hydrothermal carbonization temperature (180, 240, 300 °C) and process time (30, 90, 180 min) were summarized. Proximate and ultimate analysis, as well as low and high heating values were applied both on raw material and derived hydrochars. Additionally, the performance of the process was examined. The obtained results show that hydrothermal carbonization is a feasible method for chicken manure disposal and valorization. Although the process time did not influence the fuel properties of chicken manure considerably, a higher temperature led to a significantly higher heating value, reaching 23,880.67 ± 34.56 J × g^−1^ at 300 °C and 180 min with an improvement of ~8329 J × g^−1^ compared with raw chicken manure (15,551.67 J × g^−1^). Considering the energy gain value, the hydrochar derived at 240 °C in 30 min had the best result. Moreover, the energy consumption for this process was relatively low (124.34 ± 8.29 kJ × g^−1^). With its still feasible fuel properties and high heating value of 20,267.00 ± 617.83 kJ × g^−1^, it was concluded that these parameters of chicken manure hydrochar are the most beneficial and present a potential alternative for conventional fuel.

## 1. Introduction

### 1.1. Background

Modern poultry production and the increased associated waste, particularly excreta/manure, around the globe continue to pose a wide array of complex environmental challenges [1,2]. For instance, given the increasing global number of chickens and laying hens, the produced waste inevitably becomes challenging to tackle [1]. Poultry manure is an example of organic waste, and it becomes a challenge to appropriately manage the larger amounts being produced annually. Only Poland, which is a leader in poultry production in Europe, bred around 192.1 million birds in 2017, generating approximately 4.5 million Mg of poultry manure yearly [3,4]. The most bred poultry is chicken, and its production is responsible for generating around 20,708 million Mg of manure worldwide each year [5]. Chicken manure is a problematic waste considering its amount, as well as high moisture content, which hampers transportation and management solutions. Waste generated in poultry farms, such as droppings and litter could pose human health risks [6]. In the wake of climate changes and energy shortages, there is an increasing need for new ways of providing clean and sustainable energy [7,8]. What is more, the 17 Sustainable Development Goals (SDGs), implemented by the United Nations in 2015, are expected to be achieved by the end of 2030, with some of them referring directly to producing clean renewable energy and increasing waste recycling levels [9]. According to the International Energy Agency (IEA) [10], coal energy plants are responsible for one fifth of global greenhouse gases (GHG) emissions, representing the highest share of all sources. GHG emission is the main factor causing climate changes and global warming, leading to the increase in the mean land surface air temperature by 1.53 °C from 1850 to 2015 and it was stated that in 2019 the global mean surface temperature was 0.98 °C warmer than the 1951÷1980 period due to (GHG) emissions [11]. In this sense, creating an alternative use of poultry manure for energy production purposes while mitigating its inherent effect on the environment is warranted. For instance, the application of thermochemical conversion processes such as pyrolysis, gasification, and the direct burning of poultry manure to provide sustainable fuel [12].

### 1.2. Chicken Manure Treatment

Ways of chicken waste disposal or management include anaerobic digestion, which is the most favorable one, composting, combustion and direct land spreading [13,14]. Despite those applications, hydrothermal carbonization (HTC) may be considered a suitable method of CM management, as it is environmentally friendly in the context of emissions compared to other processes, especially composting processes, due to ammonia emissions to the atmosphere [12,15], the feasible process and the low-cost benefit over other treatments, and it is a viable way to valorize the digestate in an energy-efficient manner and at the same time maximize the synergy in terms of the recovery of water and nutrients, followed by the more efficient use of the remaining carbon [16,17]. Generally, the hydrothermal treatment process comprises different categories, namely hydrothermal carbonization or liquefaction (HTC/L) [17,18]. These types could also be classified based on operational conditions.

### 1.3. Hydrothermal Carbonization

In this work, the application of HTC was considered due to its feasible process and the low-cost benefit over pyrolysis treatment. Hydrothermal treatment is gaining popularity as one of the methods to effectively manage organic waste characterized by a high moisture content (*MC*), as the process does not require pre-drying, resulting in it being more attractive [19]. The process of HTC strongly imitates the natural processes of coal shaping [20]. It occurs in conditions of subcritical water at temperatures usually ranging from 160 to 300 °C and under autogenous pressure generated during the process of carbonization with a residence time of 30 min to several hours [8,12,17,21,22,23,24]. The mechanisms of the HTC process vary depending on the used material type; however, two of the main reactions occurring are dehydration and decarboxylation, leading to water and CO_2_ removal from the biomass material [12,17,18,19]. As a thermal valorization process, it operates effectively on biomass with high *MC* (70–90%) compared to conventional thermal technologies such as dry torrefaction and pyrolysis, which require a pre-drying process that entails additional energy usage and causes problems with odor emissions [25]. The energy requirements that are related to the production of hydrochar (*HC*), which is the main product of the HTC process, are believed to be much lower compared to those required for thermal conversion treatment with higher operating conditions.

The increases in globally generated chicken manure call for improved thermal conversion/pre-treatment-oriented solutions. The use of manure as an energy source via the HTC process for *HC* production appears promising, given the evidence of its impact of the reaction temperature on *HC* yields and the quality of chicken manure [26]. *HC* would have a higher heating value, higher energy yield, and lower ash content with respect to the same feedstock compared to biochar and the *HC* from chicken manure of 64.4% at 210 °C [27]. In addition, the treatment temperature and residence time have an influence on the composition of *HC* and the yield using poultry manure. The temperature had a significant effect leading to the enhancement of HHV by up to 25.17% at 250 °C, while the resident time had less of an impact, and ash recovery, in the *HC*, was between 74.67 and 36.59%, respectively [28]. The key properties considered of *HC* for energy purposes include mass yield, energy densification ratio, and energy yield.

### 1.4. Study Aim

The thermal transformation of waste groups still needs further studies; in fact, there appears to be a paucity of research on HTC treatment of poultry manure, specifically the impact on hydrochar’s chemical and physical properties. Such findings can also be used to process kinetic determination and to model the energy balance of HTC of the organic waste [29,30]. On this premise, the current investigation critically analyzed the HTC process of chicken manure and its energetic potential using a lab-scale pressure reactor. In particular, the specific objectives included (a) the determination of the operating conditions’ (temperature, duration, and pressure) effects on the HTC performance and (b) characterization of the fuel properties of *HC* produced from chicken manure.

## 2. Materials and Methods

### 2.1. Material (Feedstock)

Chicken manure was obtained from a farm located at the Agricultural Experiment Station Swojec that belongs to the Wroclaw University of Environmental and Life Sciences (Wroclaw, Poland). The fresh chicken manure (CM), which was up to 1 day old, was collected directly from under the cages using a metal spade and placed into two plastic buckets with a capacity of 10 L. The CM was then mixed using a drill (Bosch, model Professional GSB 16 RE, Gerlingen, Germany) with a mortar stirrer to obtain homogeneity and separated into samples of 250 g. Such prepared samples were then placed in the freezer (Electrolux, model EC5231A0W, Stockholm, Sweden) at a temperature of −27 °C until further use.

### 2.2. Methods

**Figure 1:**
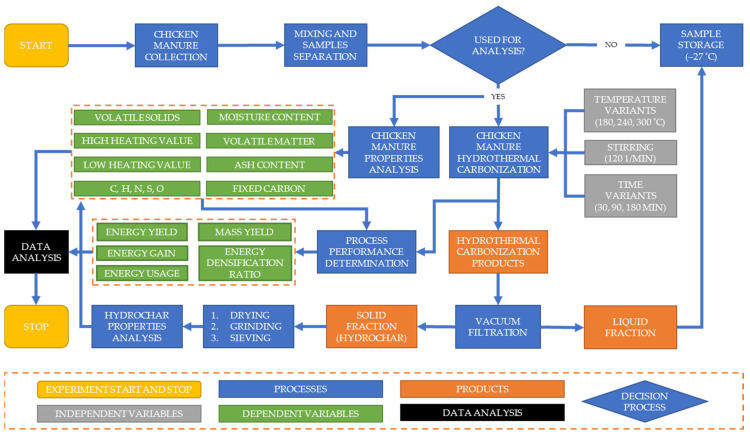
Schematic overview of the experimental procedure.

The schematic overview of the experimental procedure is presented in Figure 1.

First, chicken manure was collected and prepared for hydrothermal carbonization. Parallelly, chicken manure was subjected to fuel properties determination analyses, namely proximate analyses (moisture content, volatile matter, fixed carbon, ash content) with calorific value determination (low and high heating value) and ultimate analyses (*C*, *H*, *N*, *S*, *O*). As a result of the HTC process, hydrochar mixed with a liquid fraction was produced at various process parameters (temperature, stirring rate, and process time). During the process, energy consumption for the process was measured. After the HTC process, HTC products were separated using vacuum filtration. Next, a solid fraction known as hydrochar was prepared (drying, grinding, sieving) for the same fuel analyses as chicken manure. Afterward, using data from the HTC process and after performing fuel properties analyses, HTC performance properties were determined (mass yield, energy densification ratio, energy yield, energy gain, and energy usage). Then, all data were analyzed and the best conditions for treatment of chicken manure in the HTC process were determined. 

#### 2.2.1. HTC Process—Hydrochar Production

The HTC process was performed using a high-temperature high-pressure reactor (HPHT) (Büchi AG, Uster, Switzerland).

A sample of 220 g of CM, once thawed, was placed in the feedstock vessel, which was then placed in the heating jacket, closed, and sealed. The speed of the stirrer was set to 120 rpm and the desired temperature inside the vessel was set. The HTC processes were carried out at three different temperatures of 180, 240, and 300 °C. After reaching the temperature of 5 °C lower than the set value, the process continued for 0.5, 1.5, and 3 h (it was because of the PID temperature controller, which needs a lot of time to heat up the reactor for the last 5 °C). Each temperature was combined with each retention time three times to ensure repeatability. After the specified time elapsed, the reactor was set to cool down. During the process, the pressure was generated autogenously. Additionally, during the process, the energy consumption was recorded using an energy meter (Starmeter Instruments Co., Ltd., SK-410, Shenzhen, China). After reaching the temperature of 45 °C, the reactor was turned off, the valve was opened to release pressure and the sample was removed from the vessel using a plastic spoon. The sample was then weighed using a laboratory scale (Radwag, MA 50.R, Morawica, Poland) and the solid part was separated from the liquid by performing vacuum filtration (Rocker, ROCKER 300, Kaohsiung, Taiwan). The liquid part was placed into a plastic container and placed in the freezer (Electrolux, model EC5231A0W, Stockholm, Sweden) at a temperature of −27 °C. The solid part was weighed and placed into a laboratory dryer (WAMED, KBC-65W, Warsaw, Poland) for 24 h at 105 °C. After that time, the obtained *HC* sample was ground using an electric grinder (Royal Catering, RCMZ-800, Wuppertal, Germany) and sieved through a 0.025 mm mesh sieve to homogenize the material. Fractions below 0.025 mm were stored in plastic bags until further analyses.

#### 2.2.2. Hydrochar Fuel Properties Analyses

Raw and processed samples were tested in three replicates to ensure repeatability. The moisture content (*MC*) was determined in accordance with Świechowski et al. (2019) [31], using a laboratory dryer (WAMED, KBC-65W, Warsaw, Poland). The volatile matter content (*VM*) was measured via the thermogravimetric method by means of a tubular furnace (Czylok, RST 40 × 200/100, Jastrzębie-Zdrój, Poland), in accordance with Torquato et al. (2017) [32]. The ash content (*AC*) was determined by incinerating the sample in a muffle furnace (Snol 8.1/1100, Utena, Lithuania) in accordance with the PN-Z-15008-04:1993 standard. Fixed carbon (*FC*) was determined using the difference between VM and AC. Samples were also tested for the content of volatile solids (*VS*), according to the PN-EN 15935:2022-01 standard [33], by means of the muffle furnace (Snol 8.1/1100, Utena, Lithuania). The high heating value (*HHV*) was determined using a calorimeter (IKA, C200, Staufen, Germany), in accordance with PN EN ISO 18125:2017-07 standard [34].

The fuel ratio was calculated using Equation (1) [35].

(1) $$FR=\frac{FC}{VM}$$

where:

*FR*—fuel ratio, -;
*FC*—fixed carbon, %;
*VM*—volatile matter, %.

The low heating value (*LHV*) was calculated based on Equation (2) [3].

(2) $$LHV=HHV-\;2441.8\times \left(9\times \frac{H}{100}\right)-24.41\times \left(\frac{MC}{100}\right)\times (100-\frac{MC}{100})$$

where:

*LHV*—low heating value (as received), J × g^−1^;
*HHV*—high heating value (as dry base), J × g^−1^;
*H*—hydrogen content, %;
*MC*—moisture content, %.

The content of carbon, hydrogen, nitrogen, and sulfur elements was determined using an elemental analyzer (Perkin Elmer, 2400 Series, Waltham, MA, USA) according to the PN-EN ISO 16948:2015-07 standard [36]. The oxygen content was calculated based on Equation (3) [13].

*O* = 100% − *C* − *H* − *N* − *S* − *AC* (3)

where:

*O*—oxygen content, %;
*C*—carbon content, %;
*N*—nitrogen content, %;
*S*—sulfur content, %;
*AC*—ash content, %.

Additionally, the *H/C* and *O/C* ratios were calculated based on Equations (4) and (5) [31].

(4) $$H/C=\frac{\frac{H}{1}}{\frac{C}{12}}$$

(5) $$O/C=\frac{\frac{O}{16}}{\frac{C}{12}}$$

where:

*H/C*—molar ratio of *H* to *C*, -;
*O/C*—molar ratio of *O* to *C*, -;
1—molar mass of *H*, u;
12—molar mass of *C*, u;
16—molar mass of *O*, u.

#### 2.2.3. Hydrothermal Carbonization Performance

The mass yield (*MY*) was calculated based on Equation (6). The energy densification ratio (*EDr*) was determined using Equation (7). Energy yield (*EY*) was calculated based on Equation (8) [31].

(6) $$MY=\frac{m_{h}}{m_{r}}\times 100$$

(7) $$EDr=\frac{HHV_{h}}{HHV_{r}}\times 100$$

(8) EY=MY×EDr

where:

*MY*—mass yield, %;
*m_h_*—mass of dry hydrochar after HTC process, g;
*m_r_*—mass of dry raw material before HTC process, g;
*ED_r_*—energy densification ratio, %;
*HHV_h_*—high heating value of hydrochar after HTC process, J × g^−1^;
*HHV_r_*—high heating value of raw material before HTC process, J × g^−1^;
*EY*—energy yield, %.

Additionally, to establish the most advantageous process conditions, the energy gain (*EG*) was calculated using Equation (9) [37].

(9) $$EG=\frac{(HHV_{h}-HHV_{r})/HHV_{r}}{(m_{r}-m_{h})/m_{r}}\times 100$$

where:

*EG*—energy gain, %.

##### 2.2.4. Statistical Analysis

The two-way analysis of variance (ANOVA) with post hoc Tukey tests was performed at the level of α = 0.05 to find statistically significant differences. Statistica 13.0 software (TIBCO Software Inc., Palo Alto, CA, USA) was used for this purpose. Results obtained during statistical analysis are presented in Appendix A (Table A1, Table A2, Table A3, Table A4, Table A5, Table A6, Table A7, Table A8, Table A9, Table A10, Table A11, Table A12, Table A13, Table A14, Table A15, Table A16 and Table A17).

## 3. Results and Discussion

### 3.1. Properties of Raw Chicken Manure

The chicken manure used in this experiment contains 70.58 ± 3.65% of moisture, which proves that it is a suitable material for the HTC process. Feedstock with *MC* ranging from 75% to 90% appears to be ideal for this process [38]. The results of the proximate, ultimate, and *HHV* analyses performed on the raw material used in this study are shown in Table 1. The data were compiled with the results of studies performed by other researchers [3,39,40]. The *VM* content in CM appeared to be higher in comparison to other results. As for the *AC* content, it had an intermediate value compared to others’ results, and Hussein et al. (2017) obtained a very similar result of 21.65% of *AC* [41]. The *FC* value was lower than the value of *FC* in other CM presented in the literature, which is not a preferable result, as a high *FC* value indicates that the fuel may successfully replace conventional fossil fuels [42]. However, the *FC* content in poultry litter in general ranges from 6% to 23% [3]. Both *C* and *H* contents are similar to the ones found in the literature; however, *N* and *S* contents appear to be higher. The high amount of *N* may be caused by the high amount of protein and uric acid [43]. A high content of *S* and *N* is inadvisable in fuel as these elements are responsible for the emissions of SOx and NOx emissions which consequently pollute the environment [44,45]. The *HHV* of the studied CM was 15,551.67 ± 53.82 J × g^−1^ and was higher than the typical CM (Table 1). The same can be observed for *LHV*, which is 4232.89 ± 367.20 J × g^−1^ and is higher than the *LHV* of chicken manure *HC* obtained by Tańczuk et al. (2019) by 1031.89 J × g^−1^ [3].

### 3.2. Fuel Properties of Hydrochar

Both temperature and retention time had an influence on hydrochars’ properties, as well as on the generated pressure (Table 2); however, the temperature’s effect was more considerable. 

The *FC* content increased, while the *VM* and *VS* content reduced. The highest increase in the *FC* value was observed at 240 °C with an increase of 255.59% relative to raw CM. The highest *FC* content was obtained in *HC* derived at 300 °C at 180 min (30.33 ± 1.28%) and the result was 192.65% higher than the *FC* content in *HC* obtained at the lowest process parameters. The observed differences in *FC* content for parameters were significant (*p* < 0.05), and the same was observed for *VM* content (Table A1 and Table A2). The lowest *VM* content was present in the *HC* derived in the highest parameters (300 °C, 180 min) with a value of 37.48 ± 1.25%. The increase in the *FC* content was caused by the high temperature, which led to the devolatilization of organic matter, and therefore a higher amount of solid carbon remained in the residual volatile matter [46]. The *VS* content decreased from 78.88 ± 0.47% for raw CM to 57.92 ± 4.89% for the *HC* obtained at 300 °C in 90 min. A low *VM* content is a desirable result, as it generates a higher amount of tar, which then leads to problems with combustion or gasification systems [47]. In comparison to the *HC* obtained from different biomass feedstock, the *VM* content in this study was relatively low. For instance, *HC* derived from coconut fiber at temperatures from 220 to 375 °C, ranged from 69.8% for the lowest temperature to 42.6% for the highest temperature. As shown by Feiyue et al. (2022), *HC* derived from CM at temperatures ranging from 200 to 350 °C in 120 min, was characterized by *FC* with the highest share of 15.74% for the temperature of 300 °C [48]. Moreover, the *FC* and *VM* content in lignite accounted for 49.0% and 42.1%, respectively [48,49]. This suggests that *HC* derived from CM has beneficial fuel properties.

The highest *FR* was 0.81 ± 0.06 for *HC* derived at 300 °C in 180 min. It was observed that the fuel ratio increased with temperature and time (Table 2). Statistically significant differences among process parameters were noted (*p* < 0.05) (Table A3). As the *FR* of all obtained samples of *HC* was <2.5 (Table 2), it can be concluded that all produced hydrochars demonstrated an adequate combustion performance for pulverized fuel burning [48].

The *AC* content in fuel plays a vital role in the energy sector, as high a high content of *AC* may cause damage in furnaces, which is the effect of a high alkali metals content, and it also increases costs of waste disposal after the combustion process [48]. The *AC* increased from 21.12 ± 0.47% in raw CM to 32.20 ± 0.21% in *HC* derived at 300 °C at 180 min. This increase was also noted by other researchers, for instance, the *AC* of corn stalk increased from 4.80% for raw material to 6.12% and 12.13% for 200 °C and 250 °C, respectively [50]. The increase in the *AC* was caused by a decreased mass yield and a loss in organic material that exceeded the loss of inorganic material [50]. Additionally, the statement posed by Burra et al. (2016) [51] that CM is rich in ash when compared to other biomass sources was confirmed. For instance, the *AC* present in *HC* obtained from dead leaves at temperatures from 200 °C to 250 °C ranged from 13.64% to 21.04% [52], which is also lower than observed in this study. For watermelon peel, the *AC* ranged from 4.19% to 6.24% in *HC* derived at temperatures from 190 to 260 °C [53].

The results of elemental analysis are presented in Table 3. As expected, the *C* content increased with temperature, while the *O* content decreased.

The highest *C* content was found in *HC* derived at 300 °C in 180 min (52.23 ± 0.57%). There were statistically significant differences when compared to other process parameters. The loss in *O* content (down to 4.65 ± 1.04%) is favorable, as together with a high *C* content it leads to an energy density increase [54]. There were no considerable changes in *S* content with both time and temperature, with an average share of 1.90%. Generally, the amount of *S* should decrease as the temperature increases [53,55]; however, according to Liu et al. (2013) and Devi and Saroha (2015), the *S* amount remained stable [49,56]. *HC*s derived from different materials, e.g., *HC*s derived from beet pulp and dry leaves do not contain any *S*, regardless of temperature changes. The *N* content decreased with both time and temperature, with a decrease of 58.53% in comparison to raw material, reaching 4.85 ± 0.03% N in CM derived at the highest process parameters. However, the *N* content was also higher than in the *HC*s described above [52,55]. According to Ashworth et al. (2020), poultry manure contains essential plant nutrients, including *N* and *S*. Moreover, CM contains more *N* and *S* than other poultry manure, e.g., turkey manure [57,58]. Although these elements are not favorable in relation to combustion (as mentioned in paragraph 3.1.), it suggests that further investigation would provide additional meaningful insight into fertilizer properties of *HC* derived from CM, but also fertilizer properties of the liquid fraction which is a by-product of the HTC process.

**Figure 2:**
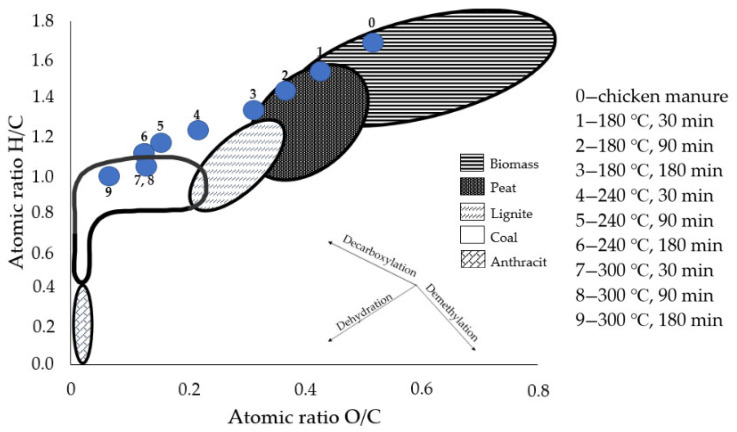
Van Krevelen’s diagram of raw and HTC treated chicken manure.

The Van Krevelen diagram (Figure 2) was used to present the fuel properties of derived *HC* by providing a comparison of the *H/C* and *O/C* ratios. Both values decreased as the process temperature increased. On the diagram, the positions of the *HC*s derived at lower parameters (as for time and temperature) were closer to the range of biomass and peat, while the *HC*s obtained at higher parameters were more similar to lignite and coal.

**Figure 3:**
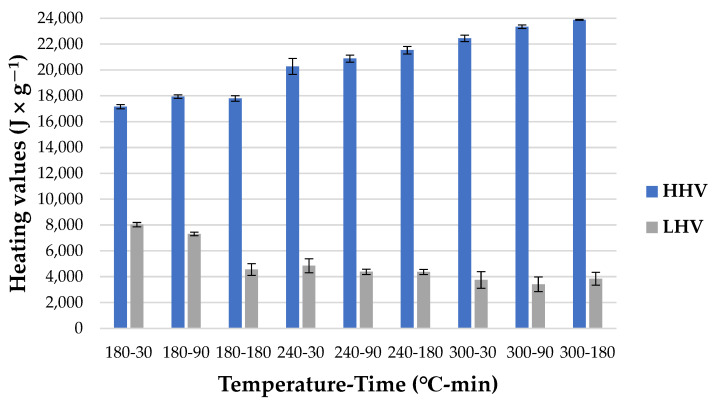
High and low heating value of hydrochars.

An *HHV* refers to the highest possible energy that is released through the full oxidation process of one fuel unit [59]. As can be seen in Figure 3, the *HHV* of *HC* increased with the increase in both temperature and time.

The *HC* derived at 300 °C in 180 min was characterized by the highest *HHV* (23,880.67 ± 34.56 J × g^−1^). The difference between this *HC* and raw CM is ~8329 J × g^−1^. The difference in HHV was statistically significantly different (*p* < 0.05) (Table A4). HTC of CM led to the production of fuel with relatively high *HHV*, as lignite is characterized by an *HHV* of ~30 MJ × kg^−1^ [48]. What is more, this value is similar to *HC*s obtained from some other types of biomass. For instance, grape pomace converted at 300 °C in 30 min reached an *HHV* of 25.29 MJ × kg^−1^, and corn stover carbonized at 300 °C in 77 min reached an *HHV* of 27,470.00 J × g^−1^. The obtained result is also higher when compared to wood materials. For instance, spruce wood and beech wood were characterized by 20.4 and 19.3 MJ × kg^−1^, respectively [60]. The *LHV* value, which allows for determining the actual energy potential of biomass [61], decreased with higher parameters of the HTC process (Figure 2), which is related to the amount of solid phase obtained during the process. The *LHV* decreased from 8030.10 ± 494.63 J × g^−1^ (180 °C-30 min) to 3410.45 ± 570.11 J × g^−1^ for *HC* obtained at 300 °C in 90 min. What is more, the *LHV* value was higher for *HC* obtained at 180 and 240 °C than raw chicken manure (4232.89 ± 367.20 J × g^−1^), whereas the *LHV* of *HC* obtained at 300 °C was lower.

### 3.3. Hydrochar and HTC Energy Yields

**Figure 4:**
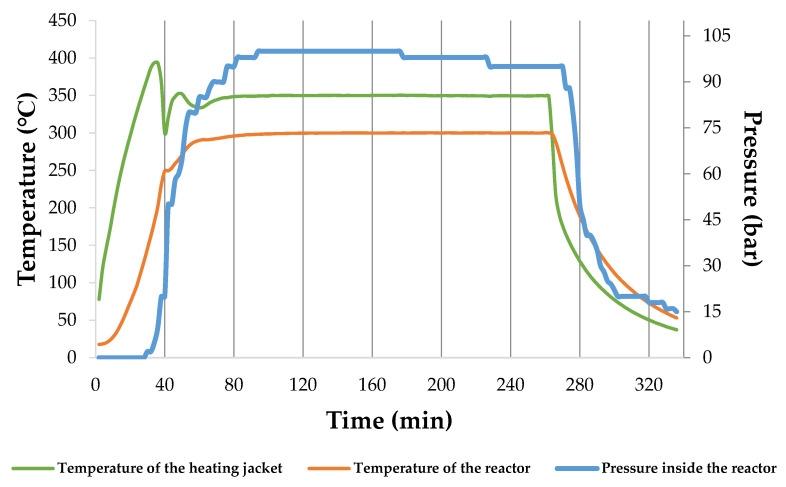
An example of temperature and pressure patterns during the process at 300 °C in 180 min.

Figure 4 shows an example of temperatures and pressure patterns during the HTC process. The average heating rate was ~4.9, ~4.0, and ~3.8 °C × min^−1^ for 180, 240, and 300 °C, respectively.

Graphs presenting temperature and pressure patterns during the HTC process with different parameters are available in Appendix B (Figure A1, Figure A2, Figure A3, Figure A4, Figure A5, Figure A6, Figure A7 and Figure A8).

**Figure 5:**
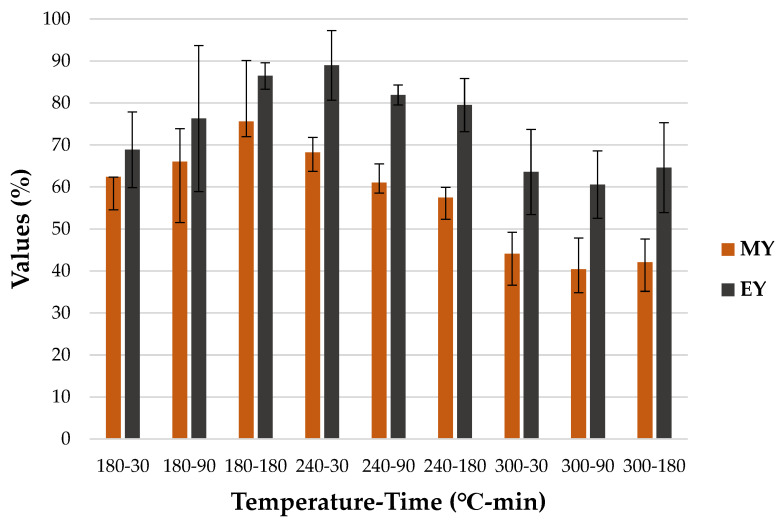
Hydrochar mass and energy yield in temperature and time.

The increase in the pressure depended on the set temperature point and the amount of produced gas, while the amount of produced gas resulted from the decomposition of the processed material. In the performed study, the retention time did not lead to a considerable *HC* yield change, which can be seen in Figure 5.

The *MY* decreased with time by 10.75 and 2.03 percentage points at 240 and 300 °C, respectively. However, in the case of the processes running at 180 °C, the *MY* increased by 13.21 percentage points with time. In the literature, both cases of increase and decrease can be found [55,62,63]. The temperature inside the reactor had a more distinctive effect on the *MY*, leading to increased mass losses. The highest *MY* was observed for *HC* derived at 180 °C in 180 min (72.25 ± 3.59%), while the *MY* was the lowest at 300 °C, generating in 90 min 40.37 ± 5.53% of the solid phase (*HC*). The observed decrease in *MY* may be induced by the decarboxylation process and the formation of organic soluble in water. As poultry manure is rich in cellulose, with an average content of 24.13%, it also leads to the decomposition and depolymerization of this component, contributing to enhanced liquid and gaseous product formation [64,65]. Similar trends were reported by other researchers [48,66]. The *HC* yield derived during the performed HTC process is higher than the *HC* yield acquired from the HTC process performed by other researchers on different kinds of feedstock. For instance, *HC* obtained from swine manure at temperatures of 200 °C and 300 °C were 55.52% and 25.64%, respectively [47]. Wheat straw subjected to the HTC process at 250 °C in 120 min reached 35.93% of *MY*. This lower yield might be caused by the composition of the compared types of biomasses, e.g., wheat straw contains more cellulose than CM, reaching between 28% and 39% [67].

**Figure 6:**
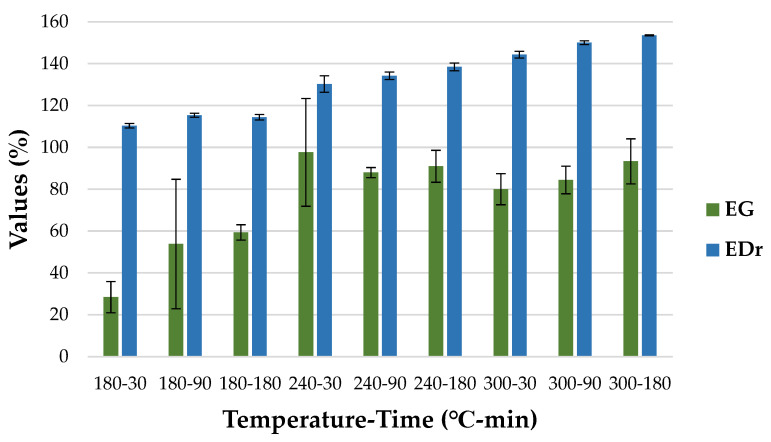
Hydrochar energy densification ratio and energy gain in temperature and time.

Energy yield is an important parameter, as it defines the amount of energy left in *HC* [49]. As presented in Figure 5, *EY* increased with time at 180 °C by 25.54%, and slightly at 300 °C by 1.62%. However, it decreased at 240 °C by 10.65%. Moreover, the *HC* obtained at 300 °C was characterized by the lowest *EY*, reaching 63.59 ± 10.13%, 60.57 ± 8.01% and 64.62 ± 10.71% in 30, 90, and 180 min, respectively. The highest *EY* was obtained from *HC* derived at 240 °C in 30 min (88.99 ± 8.29%) (Figure 5). A decrease in *EY* with increasing temperature was also noted for *HC* obtained from poultry litter, but also for biochar obtained from the same material [46]. The decreasing trend of *EY* for *HC* with increasing temperature and time is common because of material decomposition and its conversion into liquid and gas products as well [66]. The energy densification ratio shows the increase in the energy content of *HC* concerning the raw substrate. The *EDr* generally increased with time and temperature (*p* < 0.05) (Table A5) (Figure 6). The noticeable differences in standard deviation for different parameters are presented in Figure 5 and Figure 6 and might be particularly of interest for the 180-90 and 240-30 variants. This phenomenon is probably caused by the insufficient homogeneity of the samples used in the study. Chicken manure consists of droppings, bedding, and feathers. Those components are characterized by different properties. Therefore, it is possible that in some cases, prepared samples had more or less organic content than average. As result, samples with higher organic matter content showed higher decomposition and therefore lower *MY* and higher *EY*, *EG*, and *EDr* than those which had more ash content.

**Figure 7:**
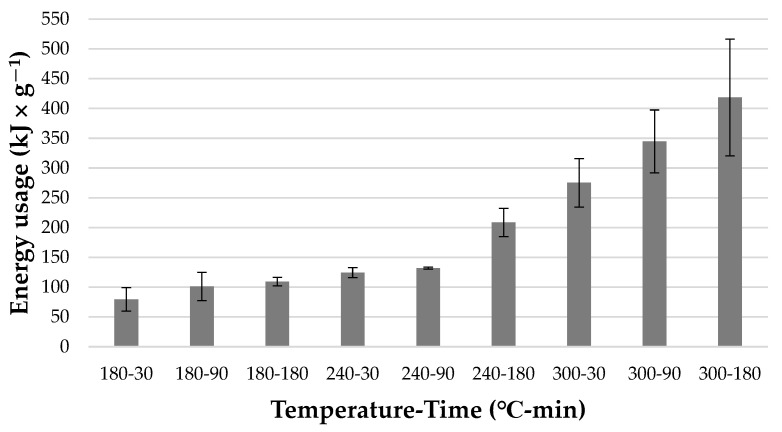
Energy usage of the HTC process in relation to the mass of dry hydrochar obtained after the process.

The highest value of *EDr* was obtained in *HC* produced at 300 °C in 180 min (153.56 ± 0.22). Generally, the *EDr* tends to increase with higher temperatures, which was proven for wheat straw, corn cob, sunflower stalk, and dry leaves [50,52]. Energy gain, which can determine the most favorable HTC conditions, shows the best compromise for mass and energy content in the derived *HC*. As shown in Figure 6, *EG* tends to increase with temperature and time. This correlation was also observed for the HTC process of eucalyptus-tree residues [37]. However, the highest *EG* was observed for *HC* obtained at 240 °C in 30 min, reaching 97.6 ± 25.78%. A statistically significant difference was observed between this *HC* and products obtained at 180 °C in 30 and 90 min (*p* < 0.05) (Table A6). This leads to the conclusion that *HC* derived at 240 °C in 30 min is more economically profitable, as it allows for the production of more energy with a slightly higher temperature and shorter time. What is more, as presented in Figure 7, energy usage (*Eu*) tends to increase with both temperature and retention time, reaching 418,270 ± 97,904 kJ × g^−1^ for *HC* obtained at 300 °C in 180 min, with a dry basis of the obtained *HC*.

The result for 240-30 was 124,336 ± 8286 kJ × g^−1^, which is 1.56 times higher than for *HC* obtained in the lowest parameters and 3.36 times lower than *HC* obtained in the highest parameters of HTC.

**Figure 8:**
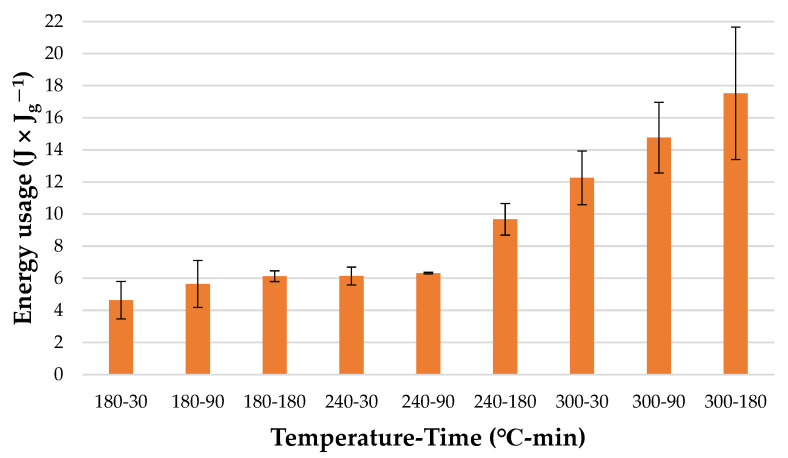
Energy usage in relation to one unit of energy available in one unit of obtained dry *HC*.

Additionally, energy usage in relation to one unit of energy available in one unit of obtained *HC* (*Eg*) was calculated (Figure 8).

The highest result was obtained for *HC* derived using the highest process parameters (300 °C, 180 min) (17.52 ± 4.12 J × J_g_^−1^). Moreover, the analysis of variance showed that there are no statistically significant differences (*p* < 0.05) (Table A7) between the amount of energy utilized for hydrochars produced at 180-30 and 240-90. There was also no significant difference between energy usage in relation to one unit of energy available in one unit of obtained *HC* for *HC* derived at 240 °C in 30 min and *HC* with the lowest energy usage (180 °C, 30 min; 4.64 ± 1.64 J × J_g_^−1^). This confirms that the most favorable conditions for the poultry manure HTC process are 240 °C and 30 min since these parameters provide the best compromise between the amount of energy left in *HC* (Figure 6) and energy usage for its production (Figure 7).

Interestingly, regardless of unit conversion, HTC energy usage for processes performed at temperatures of higher than 240 °C and residence time of 180 min are characterized by higher standard deviations than processes performed at lower temperatures and shorter residence times (Figure 7 and Figure 8). A large oscillation especially for the results at 300 °C in 180 min might be caused by the highest parameters of the process, which impacted the process and reactor behavior. A small scale of research may cause more noticeable differences. The reactor heating jacket is not well isolated from the surroundings. Therefore, at higher temperatures and longer process times, the amount of energy used to keep set temperatures inside of the reactor is more dependent on surrounding temperatures. When the temperature of the surrounding increases the energy use decreases and the opposite occurs due to higher or lower energy losses to the environment.

## 4. Conclusions

Hydrothermal carbonization was proposed as a method of chicken manure disposal with rich-energy material production at the same time. It could be a solution to problematic organic waste in terms of the amount that is generated, environmental threats and to enhance the circular economy of waste management. Both temperature and time of the process had an influence on hydrochar production and its properties; however, the impact of increasing temperature was more significant than of an increased process duration.

Both proximate and ultimate analysis led to the conclusion that hydrochar derived at the highest parameters including operating temperature and duration (that is: 300 °C, 180 min) had the best fuel characteristics. The fixed carbon, ash content and volatile matter content were 30.33 ± 1.28, 32.20 ± 0.21 and 37.48 ± 1.25%, respectively. The *H/C* and *O/C* ratios were 0.99 ± 0.02 and 0.07 ± 0.01, respectively. The high heating value reached 23,880.67 ± 34.56 J × g^−1^, and the low heating value for this hydrochar was 3850.94 ± 494.63 J × g^−1^. These results indicate that higher temperatures lead to more completed decomposition reactions and energy densification. However, the energy consumption for this process was high (418.27 ± 97.91 kJ × g^−1^ as for dry hydrochar obtained after the process), indicating that these parameters may not be efficient.

Considering the energy gain, the hydrochar derived at 240 °C in 30 min presented the best result. Moreover, the energy consumption for this process was relatively low (124.34 ± 8.29 kJ × g^−1^). Due to its feasible fuel properties and high heating value of 20,267.00 ± 617.83 kJ × g^−1^, it was concluded that these parameters of hydrothermal carbonization of chicken manure are the most beneficial. However, research conducted on a larger scale would be useful in order to minimize the high energy consumption and maximize the effectiveness of the process.

The high nitrogen and sulfur content in derived hydrochars warrants further investigation on chicken manure hydrochar fertilizer properties, but also the need to investigate the possible properties of liquid fractions obtained during the hydrothermal carbonization process as a by-product. Regarding the properties of obtained hydrochars, further investigation on the liquid part derived during the research may provide interesting results.

## Figures and Tables

**Table 1:** Results of proximate, ultimate, and heating value analysis for raw chicken manure in the current and previous studies.

Properties	CM (This Study)	Other Studies		
		CM1 [40]	CM2 [3]	CM3 [39]
Proximate Analysis (%) *				
*VM*	70.35 ± 0.23	69.23	67.50	48.79
*AC*	21.12 ± 0.47	11.64	15.60	34.70
*FC*	8.53 ± 0.38	19.13	16.90	16.51
*VS*	78.88 ± 0.47	-	-	-
Ultimate Analysis (%) *				
*C*	37.46 ± 1.19	31.54	39.67	30.04
*H*	5.22 ± 0.12	4.52	4.72	4.32
*N*	8.28 ± 0.23	3.34	5.49	3.22
*S*	1.92 + 0.09	0.56	0.40	0.35
*O*	26.00 + 2.31	60.04	34.12	29.66
**Heating value, (J × g^−1^)**				
*HHV* *	15,551.67 ± 52.82	12,980.00	13,780.00	11,950.00
*LHV*	4232.89 ± 367.20	-	3201.00	-

* as dry base.

**Table 2:** Proximate analysis and reached pressure of derived hydrochars.

Temperature (°C)	Time (min)	Pressure (Bar)	*VM* (%) *	*FC* (%) *	*FR* (-) *	*VS* (%) *	*AC* (%) *
180	30	6	67.90 ± 0.45	10.36 ± 0.79	0.15 ± 0.01	78.26 ± 0.93	21.74 ± 1.07
	90	10	67.48 ± 0.53	10.46 ± 0.26	0.16 ± 0.01	77.94 ± 0.65	22.06 ± 0.72
	180	19	61.85 ± 0.80	13.56 ± 0.86	0.22 ± 0.02	74.85 ± 1.50	24.59 ± 0.43
240	30	44	57.45 ± 1.19	14.30 ± 1.11	0.25 ± 0.02	61.77 ± 2.32	28.25 ± 0.91
	90	48	55.17 ± 0.95	13.56 ± 0.53	0.25 ± 0.01	65.37 ± 6.10	31.26 ± 0.49
	180	46	40.14 ± 0.86	27.89 ± 0.89	0.70 ± 0.04	60.00 ± 8.35	31.97 ± 0.24
300	30	103	44.22 ± 0.51	25.68 ± 0.53	0.58 ± 0.02	59.99 ± 4.99	30.10 ± 0.02
	90	98	41.91 ± 1.39	27.20 ± 1.05	0.65 ± 0.05	57.92 ± 4.89	30.89 ± 0.82
	180	101	37.48 ± 1.25	30.33 ± 1.28	0.81 ± 0.06	58.13 ± 6.66	32.20 ± 0.21

* as dry base.

**Table 3:** Elemental analysis of derived hydrochars.

Temperature (°C)	Time (min)	*C* (%) *	*H* (%) *	*N* (%) *	*S* (%) *	*O* (%) *
180	30	40.48 ± 0.23	5.20 ± 0.07	7.83 ± 0.50	1.79 ± 0.06	22.97 ± 0.59
	90	42.52 ± 0.51	5.14 ± 0.02	7.60 ± 0.36	1.93 ± 0.04	20.74 ± 0.75
	180	43.41 ± 1.45	4.89 ± 0.06	7.38 ± 0.41	1.83 ± 0.12	17.90 ± 1.72
240	30	46.09 ± 0.58	4.78 ± 0.11	5.59 ± 0.30	1.98 ± 0.05	13.31 ± 1.23
	90	47.34 ± 0.22	4.60 ± 0.11	5.40 ± 0.19	1.97 ± 0.03	9.43 ± 1.06
	180	48.19 ± 0.69	4.51 ± 0.04	5.25 ± 0.05	1.89 ± 0.08	8.12 ± 0.74
300	30	50.07 ± 0.06	4.40 ± 0.03	5.01 ± 0.03	1.92 ± 0.06	8.48 ± 0.62
	90	51.46 ± 1.51	4.38 ± 0.04	4.91 ± 0.04	1.88 ± 0.16	6.47 ± 1.25
	180	52.23 ± 0.57	4.31 ± 0.03	4.85 ± 0.03	1.87 ± 0.09	4.65 ± 1.04

* as dry base.

**Figure A1:**
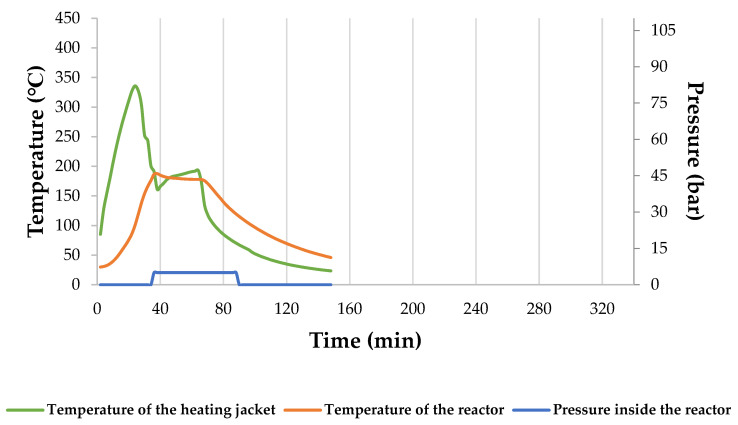
Temperature and pressure patterns during the process at 180 °C in 30 min.

**Figure A2:**
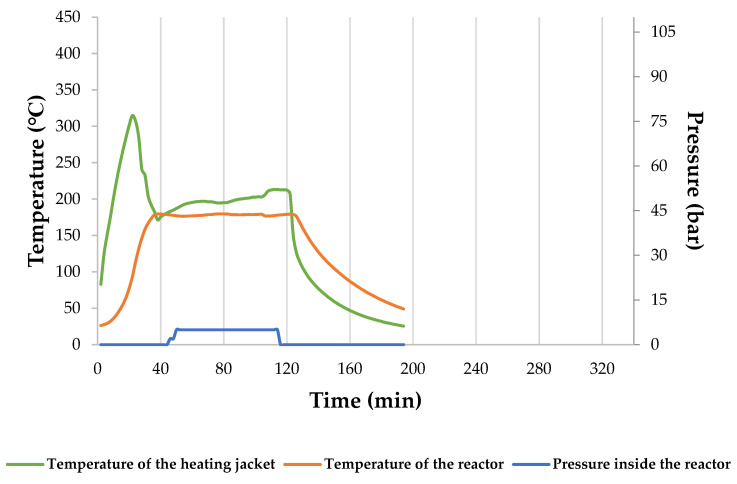
Temperature and pressure patterns during the process at 180 °C in 90 min.

**Figure A3:**
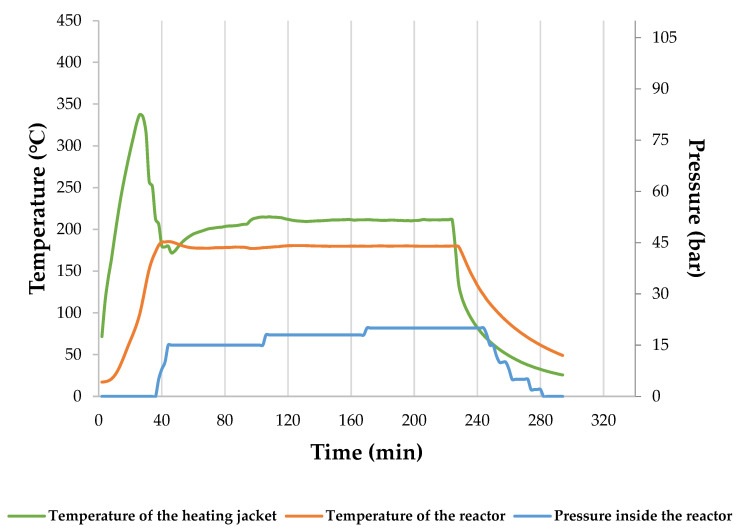
Temperature and pressure patterns during the process at 180 °C in 180 min.

**Figure A4:**
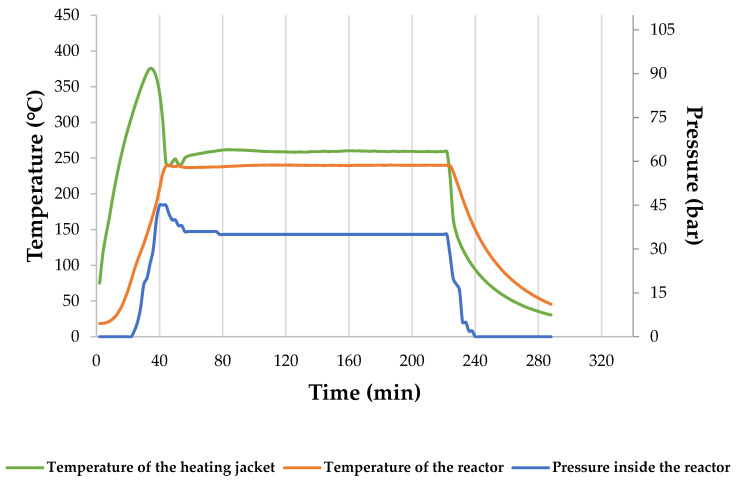
Temperature and pressure patterns during the process at 240 °C in 30 min.

**Figure A5:**
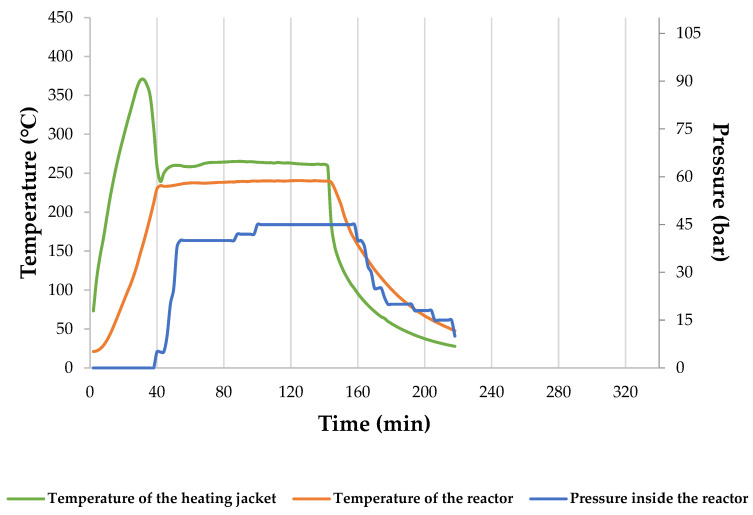
Temperature and pressure patterns during the process at 240 °C in 90 min.

**Figure A6:**
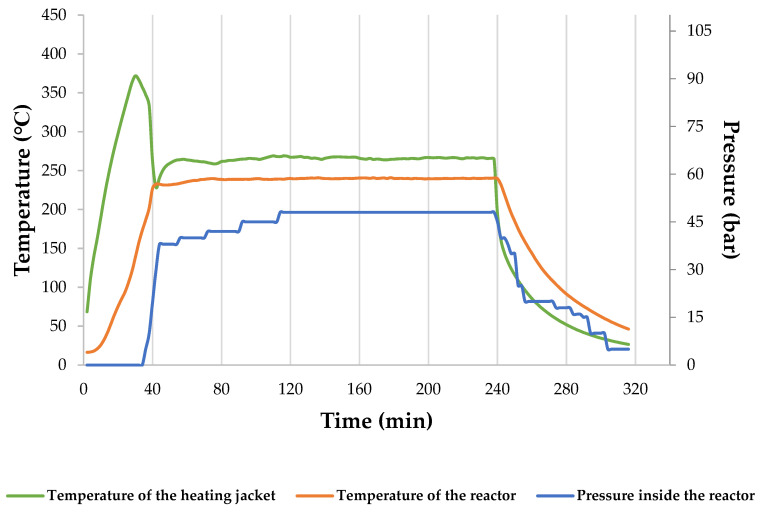
Temperature and pressure patterns during the process at 240 °C in 180 min.

**Figure A7:**
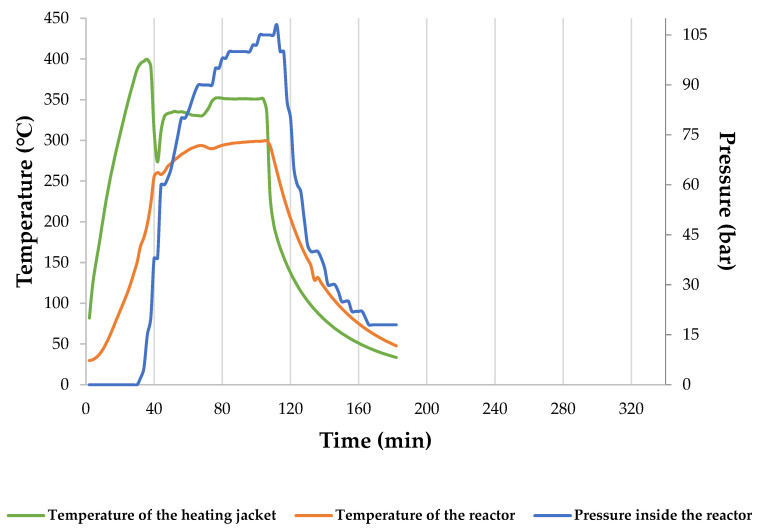
Temperature and pressure patterns during the process at 300 °C in 30 min.

**Figure A8:**
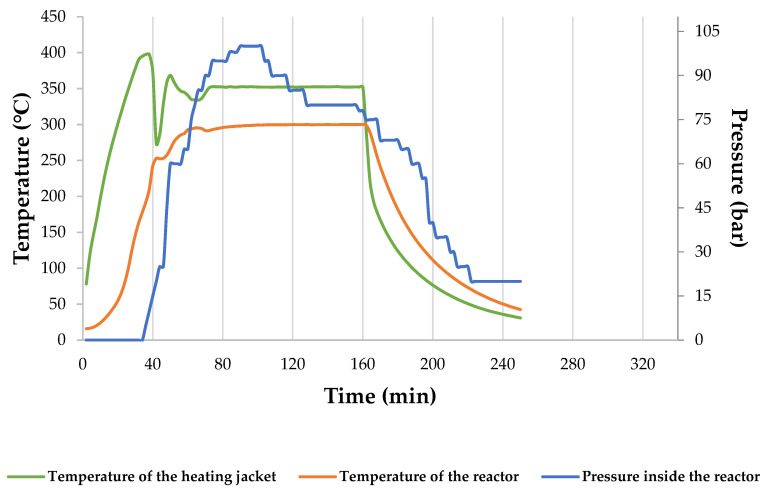
Temperature and pressure patterns during the process at 300 °C in 90 min.

## Data Availability

All data generated and used in the study are available in the article and Supplementary Materials.

## Supplementary Materials

The following supporting information can be downloaded at: https://www.mdpi.com/article/10.3390/ma15165564/s1.

## Abbreviations

*AC*	Ash content
*C*	Carbon content
CM	Chicken manure
CSF	Carbonized solid fuel
*EDr*	Energy densification ratio
*Eg*	Energy usage in relation to one unit of energy available in one unit of obtained hydrochar
*EG*	Energy gain
*EU*	Energy usage
*EY*	Energy yield
*FC*	Fixed carbon
*FR*	Fuel ratio
*H*	Hydrogen content
*H/C*	Hydrogen/carbon ratio
*HC*	Hydrochar
*HHV*	High heating value
HTC/L	Hydrothermal carbonization/liquefaction
*LHV*	Low heating value
*MC*	Moisture content
*MY*	Mass yield
*N*	Nitrogen content
*O*	Oxygen content
*O/C*	Oxygen/carbon ratio
*S*	Sulfur content
*VM*	Volatile matter
*VS*	Volatile solids

## Appendix A

Appendix A contains a statistical analysis of the results presented in the article. Bold font signifies a statistically significant difference (*p* < 0.05) between particular process parameters, e.g., 180-30 refers to the process conducted at 180 °C in 30 min.

**Table A1:** Two-way analysis of variance (ANOVA) with post hoc Tukey test for *FC* content, a bold font signifies a statistically significant difference (*p* < 0.05).

Temp-Time	180-30	180-90	180-180	240-30	240-90	240-180	300-30	300-90	300-180
180-30		1.000000	**0.002551**	**0.000402**	**0.002540**	**0.000179**	**0.000179**	**0.000179**	**0.000179**
180-90	1.000000		**0.003454**	**0.000485**	**0.003439**	**0.000179**	**0.000179**	**0.000179**	**0.000179**
180-180	**0.002551**	**0.003454**		0.954279	1.000000	**0.000179**	**0.000179**	**0.000179**	**0.000179**
240-30	**0.000402**	**0.000485**	0.954279		0.954781	**0.000179**	**0.000179**	**0.000179**	**0.000179**
240-90	**0.002540**	**0.003439**	1.000000	0.954781		**0.000179**	**0.000179**	**0.000179**	**0.000179**
240-180	**0.000179**	**0.000179**	**0.000179**	**0.000179**	**0.000179**		0.054181	0.968570	**0.007716**
300-30	**0.000179**	**0.000179**	**0.000179**	**0.000179**	**0.000179**	0.054181		0.342749	**0.000190**
300-90	**0.000179**	**0.000179**	**0.000179**	**0.000179**	**0.000179**	0.968570	0.342749		**0.001220**
300-180	**0.000179**	**0.000179**	**0.000179**	**0.000179**	**0.000179**	**0.007716**	**0.000190**	**0.001220**	

**Table A2:** Two-way analysis of variance (ANOVA) with post hoc Tukey test for *VM* content, a bold font signifies a statistically significant difference (*p* < 0.05).

Temp-Time	180-30	180-90	180-180	240-30	240-90	240-180	300-30	300-90	300-180
180-30		0.999655	**0.000177**	**0.000173**	**0.000173**	**0.000173**	**0.000173**	**0.000173**	**0.000173**
180-90	0.999655		**0.000193**	**0.000173**	**0.000173**	**0.000173**	**0.000173**	**0.000173**	**0.000173**
180-180	**0.000177**	**0.000193**		**0.000654**	**0.000174**	**0.000173**	**0.000173**	**0.000173**	**0.000173**
240-30	**0.000173**	**0.000173**	**0.000654**		0.136846	**0.000173**	**0.000173**	**0.000173**	**0.000173**
240-90	**0.000173**	**0.000173**	**0.000174**	0.136846		**0.000173**	**0.000173**	**0.000173**	**0.000173**
240-180	**0.000173**	**0.000173**	**0.000173**	**0.000173**	**0.000173**		**0.001330**	0.387176	0.053735
300-30	**0.000173**	**0.000173**	**0.000173**	**0.000173**	**0.000173**	**0.001330**		0.125784	**0.000174**
300-90	**0.000173**	**0.000173**	**0.000173**	**0.000173**	**0.000173**	0.387176	0.125784		**0.000614**
300-180	**0.000173**	**0.000173**	**0.000173**	**0.000173**	**0.000173**	0.053735	**0.000174**	**0.000614**	

**Table A3:** Two-way analysis of variance (ANOVA) with post hoc Tukey test for *FR*, a bold font signifies a statistically significant difference (*p* < 0.05).

Temp-Time	180-30	180-90	180-180	240-30	240-90	240-180	300-30	300-90	300-180
180-30		1.000000	0.059948	**0.003027**	**0.004133**	**0.000179**	**0.000179**	**0.000179**	**0.000179**
180-90	1.000000		0.075922	**0.003854**	**0.005287**	**0.000179**	**0.000179**	**0.000179**	**0.000179**
180-180	0.059948	0.075922		0.827963	0.895906	**0.000179**	**0.000179**	**0.000179**	**0.000179**
240-30	**0.003027**	**0.003854**	0.827963		1.000000	**0.000179**	**0.000179**	**0.000179**	**0.000179**
240-90	**0.004133**	**0.005287**	0.895906	1.000000		**0.000179**	**0.000179**	**0.000179**	**0.000179**
240-180	**0.000179**	**0.000179**	**0.000179**	**0.000179**	**0.000179**		**0.000613**	0.377309	**0.000237**
300-30	**0.000179**	**0.000179**	**0.000179**	**0.000179**	**0.000179**	**0.000613**		**0.047955**	**0.000179**
300-90	**0.000179**	**0.000179**	**0.000179**	**0.000179**	**0.000179**	0.377309	**0.047955**		**0.000180**
300-180	**0.000179**	**0.000179**	**0.000179**	**0.000179**	**0.000179**	**0.000237**	**0.000179**	**0.000180**	

**Table A4:** Two-way analysis of variance (ANOVA) with post hoc Tukey test for *HHV*, a bold font signifies a statistically significant difference (*p* < 0.05).

Temp-Time	180-30	180-90	180-180	240-30	240-90	240-180	300-30	300-90	300-180
180-30		0.079691	0.231818	**0.000179**	**0.000179**	**0.000179**	**0.000179**	**0.000179**	**0.000179**
180-90	0.079691		0.999219	**0.000179**	**0.000179**	**0.000179**	**0.000179**	**0.000179**	**0.000179**
180-180	0.231818	0.999219		**0.000179**	**0.000179**	**0.000179**	**0.000179**	**0.000179**	**0.000179**
240-30	**0.000179**	**0.000179**	**0.000179**		0.263009	**0.001506**	**0.000180**	**0.000179**	**0.000179**
240-90	**0.000179**	**0.000179**	**0.000179**	0.263009		0.200313	**0.000282**	**0.000179**	**0.000179**
240-180	**0.000179**	**0.000179**	**0.000179**	**0.001506**	0.200313		**0.026299**	**0.000190**	**0.000180**
300-30	**0.000179**	**0.000179**	**0.000179**	**0.000180**	**0.000282**	**0.026299**		**0.032128**	**0.001525**
300-90	**0.000179**	**0.000179**	**0.000179**	**0.000179**	**0.000179**	**0.000190**	**0.032128**		0.583350
300-180	**0.000179**	**0.000179**	**0.000179**	**0.000179**	**0.000179**	**0.000180**	**0.001525**	0.583350	

**Table A5:** Two-way analysis of variance (ANOVA) with post hoc Tukey test for *EDr*, a bold font signifies a statistically significant difference (*p* < 0.05).

Temp-Time	180-30	180-90	180-180	240-30	240-90	240-180	300-30	300-90	300-180
180-30		0.079691	0.231818	**0.000179**	**0.000179**	**0.000179**	**0.000179**	**0.000179**	**0.000179**
180-90	0.079691		0.999219	**0.000179**	**0.000179**	**0.000179**	**0.000179**	**0.000179**	**0.000179**
180-180	0.231818	0.999219		**0.000179**	**0.000179**	**0.000179**	**0.000179**	**0.000179**	**0.000179**
240-30	**0.000179**	**0.000179**	**0.000179**		0.263009	**0.001506**	**0.000180**	**0.000179**	**0.000179**
240-90	**0.000179**	**0.000179**	**0.000179**	0.263009		0.200313	**0.000282**	**0.000179**	**0.000179**
240-180	**0.000179**	**0.000179**	**0.000179**	**0.001506**	0.200313		**0.026299**	**0.000190**	**0.000180**
300-30	**0.000179**	**0.000179**	**0.000179**	**0.000180**	**0.000282**	**0.026299**		**0.032128**	**0.001525**
300-90	**0.000179**	**0.000179**	**0.000179**	**0.000179**	**0.000179**	**0.000190**	**0.032128**		0.583350
300-180	**0.000179**	**0.000179**	**0.000179**	**0.000179**	**0.000179**	**0.000180**	**0.001525**	0.583350	

**Table A6:** Two-way analysis of variance (ANOVA) with post hoc Tukey test for *EG*, a bold font signifies a statistically significant difference (*p* < 0.05).

Temp-Time	180-30	180-90	180-180	240-30	240-90	240-180	300-30	300-90	300-180
180-30		0.532195	0.297506	**0.000969**	**0.004063**	**0.002547**	**0.014402**	**0.007121**	**0.007255**
180-90	0.532195		0.999929	**0.049147**	0.199114	0.132063	0.494994	0.310076	0.226234
180-180	0.297506	0.999929		0.112451	0.387730	0.274307	0.756693	0.548802	0.404615
240-30	**0.000969**	**0.049147**	0.112451		0.996028	0.999716	0.873862	0.971603	0.999864
240-90	**0.004063**	0.199114	0.387730	0.996028		0.999999	0.998990	0.999998	1.000000
240-180	**0.002547**	0.132063	0.274307	0.999716	0.999999		0.990973	0.999748	1.000000
300-30	**0.014402**	0.494994	0.756693	0.873862	0.998990	0.990973		0.999988	0.995865
300-90	**0.007121**	0.310076	0.548802	0.971603	0.999998	0.999748	0.999988		0.999900
300-180	**0.007255**	0.226234	0.404615	0.999864	1.000000	1.000000	0.995865	0.999900	

**Table A7:** Two-way analysis of variance (ANOVA) with post hoc Tukey test for *LHV*, a bold font signifies a statistically significant difference (*p* < 0.05).

Temp-Time	180-30	180-90	180-180	240-30	240-90	240-180	300-30	300-90	300-180
180-30		0.998134	0.894723	0.881308	0.791296	0.775648	0.529865	0.491547	0.999995
180-90	0.998134		0.998684	0.998092	0.990020	0.987744	0.901792	0.877401	0.978751
180-180	0.894723	0.998684		1.000000	1.000000	0.999999	0.998487	0.997120	0.746110
240-30	0.881308	0.998092	1.000000		1.000000	1.000000	0.998960	0.997944	0.726189
240-90	0.791296	0.990020	1.000000	1.000000		1.000000	0.999928	0.999802	0.609648
240-180	0.775648	0.987744	0.999999	1.000000	1.000000		0.999957	0.999873	0.591591
300-30	0.529865	0.901792	0.998487	0.998960	0.999928	0.999957		1.000000	0.354686
300-90	0.491547	0.877401	0.997120	0.997944	0.999802	0.999873	1.000000		0.323264
300-180	0.999995	0.978751	0.746110	0.726189	0.609648	0.591591	0.354686	0.323264	

**Table A8:** Two-way analysis of variance (ANOVA) with post hoc Tukey test for *EU*, a bold font signifies a statistically significant difference (*p* < 0.05).

Temp-Time	180-30	180-90	180-180	240-30	240-90	240-180	300-30	300-90	300-180
180-30		0.999097	0.992207	0.914849	0.825810	**0.029185**	**0.000656**	**0.000178**	**0.000173**
180-90	0.999097		1.000000	0.998549	0.990130	0.099527	**0.002034**	**0.000201**	**0.000173**
180-180	0.992207	1.000000		0.999933	0.998715	0.151170	**0.003218**	**0.000222**	**0.000174**
240-30	0.914849	0.998549	0.999933		1.000000	0.310124	**0.007899**	**0.000283**	**0.000174**
240-90	0.825810	0.990130	0.998715	1.000000		0.421561	**0.012434**	**0.000344**	**0.000174**
240-180	**0.029185**	0.099527	0.151170	0.310124	0.421561		0.588943	**0.019172**	**0.000378**
300-30	**0.000656**	**0.002034**	**0.003218**	**0.007899**	**0.012434**	0.588943		0.543987	**0.012768**
300-90	**0.000178**	**0.000201**	**0.000222**	**0.000283**	**0.000344**	**0.019172**	0.543987		0.470804
300-180	**0.000173**	**0.000173**	**0.000174**	**0.000174**	**0.000174**	**0.000378**	**0.012768**	0.470804	

**Table A9:** Two-way analysis of variance (ANOVA) with post hoc Tukey test for *Eg*, a bold font signifies a statistically significant difference (*p* < 0.05).

Temp-Time	180-30	180-90	180-180	240-30	240-90	240-180	300-30	300-90	300-180
180-30		0.998482	0.979937	0.978842	0.959880	0.060731	**0.001846**	**0.000226**	**0.000174**
180-90	0.998482		0.999994	0.999992	0.999921	0.207517	**0.007184**	**0.000369**	**0.000176**
180-180	0.979937	0.999994		1.000000	1.000000	0.342616	**0.014010**	**0.000561**	**0.000179**
240-30	0.978842	0.999992	1.000000		1.000000	0.347012	**0.014269**	**0.000568**	**0.000179**
240-90	0.959880	0.999921	1.000000	1.000000		0.408394	**0.018199**	**0.000681**	**0.000181**
240-180	0.060731	0.207517	0.342616	0.347012	0.408394		0.714569	0.056654	**0.001370**
300-30	**0.001846**	**0.007184**	**0.014010**	**0.014269**	**0.018199**	0.714569		0.741379	**0.044874**
300-90	**0.000226**	**0.000369**	**0.000561**	**0.000568**	**0.000681**	0.056654	0.741379		0.644241
300-180	**0.000174**	**0.000176**	**0.000179**	**0.000179**	**0.000181**	**0.001370**	**0.044874**	0.644241	

**Table A10:** Two-way analysis of variance (ANOVA) with post hoc Tukey test for *AC*, a bold font signifies a statistically significant difference (*p* < 0.05).

Temp-Time	180-30	180-90	180-180	240-30	240-90	240-180	300-30	300-90	300-180
180-30		0.999343	**0.001621**	**0.000179**	**0.000179**	**0.000179**	**0.000179**	**0.000179**	**0.000179**
180-90	0.999343		**0.005126**	**0.000179**	**0.000179**	**0.000179**	**0.000179**	**0.000179**	**0.000179**
180-180	**0.001621**	**0.005126**		**0.000253**	**0.000179**	**0.000179**	**0.000179**	**0.000179**	**0.000179**
240-30	**0.000179**	**0.000179**	**0.000253**		**0.000978**	**0.000236**	0.060754	**0.003446**	**0.000304**
240-90	**0.000179**	**0.000179**	**0.000179**	**0.000978**		0.913416	0.470757	0.998303	0.833690
240-180	**0.000179**	**0.000179**	**0.000179**	**0.000236**	0.913416		0.055773	0.559710	0.999991
300-30	**0.000179**	**0.000179**	**0.000179**	0.060754	0.470757	0.055773		0.855566	0.058638
300-90	**0.000179**	**0.000179**	**0.000179**	**0.003446**	0.998303	0.559710	0.855566		0.484494
300-180	**0.000179**	**0.000179**	**0.000179**	**0.000304**	0.833690	0.999991	0.058638	0.484494	

**Table A11:** Two-way analysis of variance (ANOVA) with post hoc Tukey test for *C*, a bold font signifies a statistically significant difference (*p* < 0.05).

Temp-Time	180-30	180-90	180-180	240-30	240-90	240-180	300-30	300-90	300-180
180-30		0.227399	**0.027356**	**0.000198**	**0.000180**	**0.000179**	**0.000179**	**0.000179**	**0.000179**
180-90	0.227399		0.953385	**0.005171**	**0.000360**	**0.000195**	**0.000179**	**0.000179**	**0.000179**
180-180	**0.027356**	0.953385		0.051131	**0.002111**	**0.000380**	**0.000180**	**0.000179**	**0.000179**
240-30	**0.000198**	**0.005171**	0.051131		0.774741	0.201874	**0.001880**	**0.000217**	**0.000258**
240-90	**0.000180**	**0.000360**	**0.002111**	0.774741		0.963841	**0.045488**	**0.001348**	**0.001602**
240-180	**0.000179**	**0.000195**	**0.000380**	0.201874	0.963841		0.312711	**0.011444**	**0.010982**
300-30	**0.000179**	**0.000179**	**0.000180**	**0.001880**	**0.045488**	0.312711		0.671354	0.504387
300-90	**0.000179**	**0.000179**	**0.000179**	**0.000217**	**0.001348**	**0.011444**	0.671354		0.999892
300-180	**0.000179**	**0.000179**	**0.000179**	**0.000258**	**0.001602**	**0.010982**	0.504387	0.999892	

**Table A12:** Two-way analysis of variance (ANOVA) with post hoc Tukey test for *H*, a bold font signifies a statistically significant difference (*p* < 0.05).

Temp-Time	180-30	180-90	180-180	240-30	240-90	240-180	300-30	300-90	300-180
180-30		0.982533	**0.001958**	**0.000225**	**0.000179**	**0.000179**	**0.000179**	**0.000179**	**0.000179**
180-90	0.982533		**0.013231**	**0.000551**	**0.000180**	**0.000179**	**0.000179**	**0.000179**	**0.000179**
180-180	**0.001958**	**0.013231**		0.711229	**0.005572**	**0.000477**	**0.000185**	**0.000182**	**0.000182**
240-30	**0.000225**	**0.000551**	0.711229		0.157640	**0.010655**	**0.000419**	**0.000305**	**0.000267**
240-90	**0.000179**	**0.000180**	**0.005572**	0.157640		0.874698	0.079903	**0.043027**	**0.016201**
240-180	**0.000179**	**0.000179**	**0.000477**	**0.010655**	0.874698		0.647226	0.454186	0.174881
300-30	**0.000179**	**0.000179**	**0.000185**	**0.000419**	0.079903	0.647226		0.999993	0.951913
300-90	**0.000179**	**0.000179**	**0.000182**	**0.000305**	**0.043027**	0.454186	0.999993		0.991288
300-180	**0.000179**	**0.000179**	**0.000182**	**0.000267**	**0.016201**	0.174881	0.951913	0.991288	

**Table A13:** Two-way analysis of variance (ANOVA) with post hoc Tukey test for *N*, a bold font signifies a statistically significant difference (*p* < 0.05).

Temp-Time	180-30	180-90	180-180	240-30	240-90	240-180	300-30	300-90	300-180
180-30		0.992151	0.749174	**0.000181**	**0.000180**	**0.000179**	**0.000179**	**0.000179**	**0.000179**
180-90	0.992151		0.994697	**0.000191**	**0.000182**	**0.000180**	**0.000179**	**0.000179**	**0.000180**
180-180	0.749174	0.994697		**0.000261**	**0.000194**	**0.000183**	**0.000180**	**0.000180**	**0.000181**
240-30	**0.000181**	**0.000191**	**0.000261**		0.997828	0.920371	0.455796	0.271316	0.288165
240-90	**0.000180**	**0.000182**	**0.000194**	0.997828		0.999528	0.855040	0.655387	0.634350
240-180	**0.000179**	**0.000180**	**0.000183**	0.920371	0.999528		0.990572	0.927863	0.896584
300-30	**0.000179**	**0.000179**	**0.000180**	0.455796	0.855040	0.990572		0.999981	0.999587
300-90	**0.000179**	**0.000179**	**0.000180**	0.271316	0.655387	0.927863	0.999981		1.000000
300-180	**0.000179**	**0.000180**	**0.000181**	0.288165	0.634350	0.896584	0.999587	1.000000	

**Table A14:** Two-way analysis of variance (ANOVA) with post hoc Tukey test for *S*, a bold font signifies a statistically significant difference (*p* < 0.05).

Temp-Time	180-30	180-90	180-180	240-30	240-90	240-180	300-30	300-90	300-180
180-30		0.637097	0.999163	0.315181	0.396858	0.920547	0.757102	0.920547	0.999774
180-90	0.637097		0.932724	0.999490	0.999958	0.999490	1.000000	0.999490	0.955744
180-180	0.999163	0.932724		0.661863	0.757102	0.998679	0.975403	0.998679	1.000000
240-30	0.315181	0.999490	0.661863		1.000000	0.953319	0.995645	0.953319	0.751432
240-90	0.396858	0.999958	0.757102	1.000000		0.980704	0.999163	0.980704	0.827812
240-180	0.920547	0.999490	0.998679	0.953319	0.980704		0.999981	1.000000	0.999078
300-30	0.757102	1.000000	0.975403	0.995645	0.999163	0.999981		0.999981	0.984060
300-90	0.920547	0.999490	0.998679	0.953319	0.980704	1.000000	0.999981		0.999078
300-180	0.999774	0.955744	1.000000	0.751432	0.827812	0.999078	0.984060	0.999078	

**Table A15:** Two-way analysis of variance (ANOVA) with post hoc Tukey test for *O*, a bold font signifies a statistically significant difference (*p* < 0.05).

Temp-Time	180-30	180-90	180-180	240-30	240-90	240-180	300-30	300-90	300-180
180-30		0.211682	**0.000410**	**0.000179**	**0.000179**	**0.000179**	**0.000179**	**0.000179**	**0.000179**
180-90	0.211682		0.055413	**0.000180**	**0.000179**	**0.000179**	**0.000179**	**0.000179**	**0.000179**
180-180	**0.000410**	0.055413		**0.000930**	**0.000179**	**0.000179**	**0.000179**	**0.000179**	**0.000179**
240-30	**0.000179**	**0.000180**	**0.000930**		**0.004665**	**0.000386**	**0.000624**	**0.000181**	**0.000180**
240-90	**0.000179**	**0.000179**	**0.000179**	**0.004665**		0.834830	0.960509	**0.042917**	**0.003594**
240-180	**0.000179**	**0.000179**	**0.000179**	**0.000386**	0.834830		0.999979	0.506188	0.051070
300-30	**0.000179**	**0.000179**	**0.000179**	**0.000624**	0.960509	0.999979		0.306933	**0.026388**
300-90	**0.000179**	**0.000179**	**0.000179**	**0.000181**	**0.042917**	0.506188	0.306933		0.769687
300-180	**0.000179**	**0.000179**	**0.000179**	**0.000180**	**0.003594**	0.051070	**0.026388**	0.769687	

**Table A16:** Two-way analysis of variance (ANOVA) with post hoc Tukey test for *MY*, a bold font signifies a statistically significant difference (*p* < 0.05).

Temp-Time	180-30	180-90	180-180	240-30	240-90	240-180	300-30	300-90	300-180
180-30		0.999311	0.452863	0.984550	1.000000	0.994656	0.125802	**0.039934**	0.085584
180-90	0.999311		0.802487	0.999988	0.993907	0.873134	**0.040730**	**0.012082**	**0.030419**
180-180	0.452863	0.802487		0.939617	0.336426	0.130783	**0.001864**	**0.000656**	**0.001932**
240-30	0.984550	0.999988	0.939617		0.948429	0.692900	**0.020329**	**0.005975**	**0.016267**
240-90	1.000000	0.993907	0.336426	0.948429		0.999432	0.184553	0.061290	0.122765
240-180	0.994656	0.873134	0.130783	0.692900	0.999432		0.440551	0.176630	0.291614
300-30	0.125802	**0.040730**	**0.001864**	**0.020329**	0.184553	0.440551		0.999223	0.999732
300-90	**0.039934**	**0.012082**	**0.000656**	**0.005975**	0.061290	0.176630	0.999223		1.000000
300-180	0.085584	**0.030419**	**0.001932**	**0.016267**	0.122765	0.291614	0.999732	1.000000	

**Table A17:** Two-way analysis of variance (ANOVA) with post hoc Tukey test for *EY*, a bold font signifies a statistically significant difference (*p* < 0.05).

Temp-Time	180-30	180-90	180-180	240-30	240-90	240-180	300-30	300-90	300-180
180-30		0.985620	0.413447	0.261108	0.752136	0.895446	0.998506	0.971773	0.996339
180-90	0.985620		0.915607	0.775833	0.997661	0.999960	0.776006	0.550693	0.779922
180-180	0.413447	0.915607		0.999994	0.999482	0.990366	0.146593	0.073108	0.187361
240-30	0.261108	0.775833	0.999994		0.989291	0.941198	0.082249	**0.039490**	0.113846
240-90	0.752136	0.997661	0.999482	0.989291		0.999996	0.364845	0.203647	0.408015
240-180	0.895446	0.999960	0.990366	0.941198	0.999996		0.535524	0.326783	0.565892
300-30	0.998506	0.776006	0.146593	0.082249	0.364845	0.535524		0.999976	1.000000
300-90	0.971773	0.550693	0.073108	**0.039490**	0.203647	0.326783	0.999976		1.000000
300-180	0.996339	0.779922	0.187361	0.113846	0.408015	0.565892	1.000000	1.000000	

## Appendix B

Appendix B contains graphs presenting temperature and pressure patterns during the HTC process with different parameters.

Figure A1, Figure A2 and Figure A3 present the temperature and pressure patterns for the HTC process performed at 180 °C for 30, 90 and 180 min, respectively.

Figure A4, Figure A5 and Figure A6 present the temperature and pressure patterns for the HTC process performed at 240 °C for 30, 90 and 180 min, respectively.

Figure A7 and Figure A8 present the temperature and pressure patterns for the HTC process performed at 300 °C for 30, 90 min, respectively.
